# Nonmedical Abuse of Benzodiazepines in Opiate-Dependent Patients in Tehran, Iran

**Published:** 2012

**Authors:** Masuade Babakhanian, Maliheh Sadeghi, Nader Mansoori, Zahra Alam Mehrjerdi, Mahmood Tabatabai

**Affiliations:** 1 Department of Social Work, Baradaran e Rezaee Hospital, Semnan University of Medical Sciences, Damghan, Iran.; 2 Faculty of Management and Information, Tehran University of Medical Sciences, Tehran, Iran.; 3 Shahid Beheshti University, Family Research Institute, Tehran, Iran.; 4 Iranian National Center for Addiction Studies (INCAS), Tehran University of Medical Sciences, Tehran, Iran.; 5 Department of Psychology, Faculty of Psychology and Educational Sciences, Ferdowsi University, Mashhad, Iran

**Keywords:** Benzodiazepines abuse, Nonmedical abuse, Opiate dependence, Treatment

## Abstract

**Objective:** The purpose of the present preliminary study was to explore the prevalence of nonmedical abuse of benzodiazepines in a group of opiate-dependent patients who were on methadone maintenance treatment (MMT) program in outpatient clinics in the south-west of Tehran, Iran.

**Methods:** 114 male and female opiate-dependent clients who met DSM.IV-TR criteria for opiate dependence with mean age 36.5 years participated in the study from 16 clinics and completed a self-report questionnaire on demographics and substance use details. Then the participants were interviewed on the details of nonmedical abuse of benzodiazepines.

**Results:** The study findings indicated that the current nonmedical abuse of benzodiazepines was commonly prevalent among participants. The most common current benzodiazepines abused were alprazolam (100%) followed by chlordiazepoxide (96.5%), clonazepam (94.7%), diazepam (86.8%), lorazepam (79.8%) and oxazepam (73.7%) respectively. Depression (77%) and anxiety (72.8%) were frequently reported as the most important reasons associated with consuming benzodiazepines followed by problem in anger control (44.7%), suicide thought (12.3%), self-injury (7.9%), and suicide commitment (5.3%) respectively.

**Conclusion:** Nonmedical abuse of benzodiazepines is an important problem among opiate addicts which should be considered in treatment interventions during MMT program.

## Introduction

Nonmedical abuse of benzodiazepines is a prevalent problem among opiate-dependent individuals in Iran which could be associated with precipitating illicit opiate abuse and dependence but it has received a little attention in treatment programs of opiate addicts in this country. 

Nonmedical abuse of benzodiazepines is defined as any abuse or non-medical use of a benzodiazepine that is, the use of a drug for something other than its medical or psychiatric purpose. 

Despite the effectiveness for treating symptoms of different conditions, the increase in prescription rates for benzodiazepine anxiolytics has dramatically raised public health concerns because of the high degree of abuse potential of these medications (1).

*The Monitoring the Future Study* (MTF) examines a nationally representative sample of U.S. high school students each year and tracks a sub-sample following high school. This study found that there has been a steady increase in the nonmedical use of benzodiazepine anxiolytics among college students aged 19-22 and past year nonmedical use has more than doubled between 1994 and 2001 (1.8% and 5.1%, respectively) (2).

A study in the U.S.A employing a large sample population to examine the prevalence rate of nonmedical abuse of benzodiazepines found that 1 in 10 individuals reported the misuse of a benzodiazepine at some time in his/her lifetime (3).

A study on colleague students also in USA showed that nonmedical use of benzodiazepines was more likely to occur among college students who were White, had both male and female sex partners and reported higher rates of substance use and other risky behaviors (4).

Studies show that some individuals may have nonmedical abuse of benzodiazepines because they believe that they are less harmful compared with illicit drugs, or because they may cost less than common illicit drugs. A study in Canada has shown that up to 20% of individuals over the age of 60 obtain long-term prescriptions for pain medications (5).

*The Drug Abuse Warning Network* (DAWN) also suggests that there has been a recent increase in the abuse of prescription benzodiazepine anxiolytics. DAWN, a national surveillance system that monitors trends in drug-related emergency department (ED) visits and deaths, serves as an indicator of the harmful consequences associated with the nonmedical use of benzodiazepine anxiolytics. According to DAWN report, ED mentions of benzodiazepines dramatically increased (38%) from 1995 to 2002 in particular, including alprazolam and clonazepam (6).

Social, psychologic, motivational, and demographic factors can be associated with different forms of prescription drug misuse (7). In different studies on addiction, the observed heterogeneity among substance users has increased numerous investigations of individual difference factors relating to drug and alcohol use. These efforts have provided significant scientific support for relationships between various personality characteristics, motivations for substance use, and development of substance use problems (8).

 Among the studies that have limited their samples to individuals who report use of such drugs outside the confines of a physician's prescription, associations have been found between use of nonprescribed anxiolytics or sedatives and increased levels of psychiatric symptomatology, substance use, and other risky behaviors (9).

Despite all of these studies on general population, study on the prevalence of nonmedical abuse of benzodiazepines among opiate-dependent individuals is still subject to a paucity of research not only in the world but also in Iran while constant abuse of these drugs may easily result in severe drug dependence and addiction.

Of the few Iranian studies on this issue, Naranjiha and colleagues (2008) in their rapid situation assessment of substance abuse and dependence showed that more than half of the drug addicts (55.4%) in their study had a history of benzodiazepines abuse. Their study results also showed that alprazolam (24.5%), lorazepam (23.2%) and oxaspam (%11.4) abuse were the most prevalent drugs abused (10).

Benzodiazepines can be consumed for many reasons; however, drugs with psychotropic properties are usually abused by many abusers of illicit drugs. Development and increased availability of benzodiazepines have significantly improved treatment of pain, and mental health conditions such as anxiety, and other mental health conditions. Many individuals use benzodiazepines safely and responsibly. However, increased availability and variety of medications with psychoactive effects have contributed to benzodiazepine abuse and substantially addiction. There is a paucity of research on the prevalence of nonmedical abuse of benzodiazepines among opiate-dependent patients while chronic abuse of these medications could result in addiction. The present study was designed to examine the prevalence of nonmedical abuse of benzodiazepines in a sample of male and female opiate-dependent patients referring to outpatient MMT clinics in the south-west of Tehran, Iran.

## Materials and Methods

*Participants*

Men and women aged 18-50, who met DSM-IV.TR criteria for opiate dependence (11) within the past 12 months before treatment entry and were on Methadone Maintenance Treatment (MMT) were eligible to participate. Treatment-seeking participants were recruited through referrals from 16 MMT clinics and through poster demonstrations in MMT clinics that agreed to cooperate with us. Participation was voluntary and confidential.

In this way, 114 treatment seekers (99 males and 15 females) with mean age of 36.5(SD=10.5) years participated in the study.

Inclusion criteria included being opiate abuser, being in treatment for at least 2 weeks and signing consent form for entry to the study on the interview day. Exclusion criteria included a history of or current neurological, severe psychiatric and/or psychotic disorders which could interfere with the interview procedure.

The study was approved by the institutional review board of Tehran University of Medical Sciences. All participants provided written informed consent after being fully informed of potential benefits of participation.

*Study Design*

The interview procedure was conducted in a quiet room in each clinic during winter 2009. After giving informed consent, potential participants were screened using the MINI International Neuropsychiatric Interview (12), a structured interview for assessment of psychiatric and substance use symptoms. First demographics and details of substance use were completed for each participant. Then they were interviewed on details of nonmedical use of benzodiazepines based on a questionnaire designed by the researchers of the study.

*Statistical Analysis*

*Data were analyzed by SPSS software (*version 16.0*), using descriptive indices such as mean and its standard deviation (SD), frequency, and percentage to express data*.

## Results

The majority of the participants were male (86.8%) and the remaining were female (13.2%). The mean age of the participants was 36.5±10.5 years. The age range was 20-48 years old. Most of them were married (52.6%) and had 12 years of formal education or less. At the time of study, 30.7% of them were unemployed. Meanwhile, the mean months of presence in the current treatment was 8.1±6.4 months. All participants were opiate abusers before entry to current MMT treatment and the main routes of opiate use were smoking (85.6%) and injecting (14.03%) respectively. Duration of opiate addiction was 7±7.2 years, and average days of opiate abuse within the past month before treatment entry were 29.1 (SD=3.2) days (See details of demographics and opiate abuse of the participants in table 1).

**Table1:** Demographic characteristics of the participants (*n=*114)

**Variable**			** N / (** **٪** **)**
Gender	Male		99 (86.8%)
	Female		15 (13.2%)
		
Mean age (Year)			36.5 (SD=10.5)
Age range (Year)			20-48
Marital status	Married		
			60 (52.6%)
	Single		41 (36%)
	Divorced		8 (7%)
	Separated		3 (2.6%)
	Widow/widower		2 (1.8%)
Educational status	Less than 12 years		
			34 (29.8%)
	12 years		64 (57.7%)
	More than 12 years		16 (14%)
Employment	Employed		
			69 (60.5%)
	Unemployed		35 (30.7%)
	Homemaker		10 (8.7%)
		
Opiate abuse before treatment entry			114 (100%)
		
Main route of opiate use		smoking	98 (85.6%)
		Injecting	16 (14.03%)
		
Duration of opiate dependence (Years)			7 (SD=7.2)
Mean days of opiate use in the last month before treatment entry			29 (SD=3.2)

The study findings showed that nonmedical abuse of benzodiazepines was prevalently common among the participants. Participants frequently reported that they abused benzodiazepines without formal prescription. Mean onset age of nonmedical abuse of benzodiazepines was 27.6±9.9 years and the duration of abuse was 4.7±6.1 years indicating that participants initiated consuming nonmedical abuse of benzodiazepines in the first years of young adulthood and became chronic abusers for almost a long time. Average days of benzodiazepines abuse were 18.4±10.9 days and they consumed a variety of benzodiazepines within the past 12 months before entry to MMT program. All participants had experience with consuming alprazolam (100%) followed by consuming chlordiazepoxide HCL (96.5%), clonazepam (94.7%), diazepam (86.8%), lorazepam (79.8%), and oxaspam (73.7%) respectively before entry to MMT program.

Participants had also the history of abusing other substances in their lives such as opium, heroin, crystalline heroin, hashish, methamphetamine, and alcohol (See details in table 2).

**Table2:** History of drug abuse of the participants (*n=*114)

**Drug of abuse**	**Frequency(Percentae)**
**Benzodiazepines**	
Alprazolam	114 (100%)
Chlordiazepoxide HCL	110 (96.5%)
Clonazepam	108 (94.7%)
Diazepam	99 (86.8%)
Lorazepam	91 (79.8%)
Oxazepam	84 (73.7%)
	
Mean onset age of benzodiazepines initiation	27.6 (SD=9.9)
	
Duration of benzodiazepines abuse	4.7 (SD=6.1)
	
Average days of benzodiazepines abuse	18.4 (SD=10.9)
	
**Other substances**	
Opium	111 (97.4%)
Heroin	38 (33.3%)
Crystalline heroin	57 (50%)
Hashish (cannabis)	77 (76.5%)
Methamphetamine	64 (56.1%)
Alcohol	92 (80.7%)

Participants reported a wide range of experiencing affective problems within the past 6 months before entry to MMT programs that resulted in consumption of benzodiazepines. Among these conditions, depression (77%) and anxiety (72.8%) were frequently reported as reasons associated with consuming benzodiazepines followed by problem in anger control, suicide thought, self-injury, and commitment. A few of the participants (7%) had also history of outpatient treatment for affective disorders and one participant had inpatient treatment history for treating affective disorders (Table 3).

**Table3:** Profile of current affective problems among the participants (*n=*114)

**Variable**	**N / (** **٪** **)**
Depression	84(77%)
Anxiety	83(72.8%)
Anger control	51(44.7%)
Suicide thought	14(12.3%)
Self-injury	9(7.9%)
Suicide commitment	6(5.3%)
Outpatient treatment for affective disorders	8(7%)
Inpatient treatment for affective disorders	1(0.09%)

## Discussion

Benzodiazepines have important applications in the treatment of anxiety and sleep disorders. Furthermore, addition to having beneficial clinical effects, these drugs have been shown to be vulnerable to misuse (13). The trend in prescription rates of such medications is related to the discussion of prescription drug abuse because an increase in prescription rates may accelerate the likelihood that these medications will be abused (14).

The present study found that nonmedical abuse of benzodiazepines was significantly prevalent among opiate dependent clients that participated in the study. Participants in the study reported that they consumed benzodiazepines before opiate use initiation and commonly before entry to MMT program.

The present study findings are consistent with the study findings of Esteban McCabe. In his study on US college students, at individual and college levels, the prevalence of nonmedical benzodiazepines use co-occurred to a high degree with the nonmedical use of prescription opiates (15). In his study, he found 65.7% of nonmedical benzodiazepine users also used prescription opiate analgesics no medically in the past year before entry to study as compared to only 4.4% of students who did not use benzodiazepines for nonmedical purposes (15).

One of the important findings in our study was this fact that participants in the study were ploy drugs. They consumed opiates such as opium, heroin and crystalline heroin with benzodiazepines. The pattern of polydrug use among the participants found in the present study is consistent with previous findings showing the nonmedical use of benzodiazepines occurs largely among people who use other drugs (16).

Considering the prevalence of nonmedical use of benzodiazepines among opiate-dependent patients, it is imperative to continue monitoring this drug use behavior over time and to develop and evaluate prevention programs aimed at reducing prescription drug abuse. There is a clear need to balance medical necessity of benzodiazepines and basically the risk of nonmedical use and abuse of these drugs (17).

This study provides evidence that nonmedical use of benzodiazepines represents a problem among some opiate-dependent individuals in Iran. These findings have important implications for developing prevention efforts aimed at reducing the nonmedical use of benzodiazepines among opiate abusers while not hindering the effective clinical treatment for various reasons underlying this important issue such as anxiety disorders and other affective disorders including depression and suicide thought which were prevalent among the participants in this study. Indeed, stressful conditions were significant predictors for abusing benzodiazepines which could precipitate dependence on substances with opiate among the participants. This study finding was consistent with other studies (14).

Longitudinal research is still necessary to examine the relationship between nonmedical use of benzodiazepines and development of substance use disorders (14).

Despite the high prevalence and recent increases in nonmedical use of benzodiazepines, these drugs have remained as highly effective and safe for the majority of patients (16). However, findings from the present study provide strong support for the hypothesis that the nonmedical use of sedative-hypnotics like benzodiazepines represents a problem among certain segments of the opiate-using population. This issue needs to be deterred with effective prevention efforts and therapeutic strategies while not hindering effective clinical treatment of affective disorders.

Several limitations need to be considered when evaluating the study’s findings. First, the present study was subject to the limitations of self-reports. However, such studies have been widely used and are considered valid in examining drug use when certain conditions of confidentiality are met (18, 19). For instance, participation in the study was voluntary, the reasons of conducting the study was explained, and respondents were assured that their responses would remain anonymous. Furthermore, our study was limited to one area of Tehran so the results may not be generalizable to other groups of opiate abusers in other parts of Tehran and it is subject to further studies with larger sample populations and including other areas of Tehran

## Authors’ Contributions

MB and ZAM conceived and designed the evaluation and helped to draft the manuscript. MS and NM participated in designing the evaluation and performed the statistical analysis. MT collected the clinical data and re-evaluated them. All authors read and approved the final manuscript.

## Notes

**Declaration of Interest:** None.
